# Abortion in Cows1Presented at the annual meeting of the Pennsylvania State Veterinary Medical Association, March 4 and 5, 1902.

**Published:** 1902-06

**Authors:** Walter S. Phillips

**Affiliations:** Reading, Pa.


### ABORTION IN COWS.[1]

By Walter S. Phillips, V.S.,

READING, PA.

Abortion is an affection which, when once it affects the farmer's or dairyman's herd, causes much vexation and loss.

Abortion is so called when the fœtus or impregnated ovum is expelled four or six weeks before the normal period; premature birth, that is, when the fœtus is advanced enough to live. Mares are also subject to it, but very few of them.

I notice a great veterinarian has said that abortion is a disease of nervous origin—a loss of equilibrium between the nerves of voluntary and involuntary motion. The direct cause of this state exists in anything that can derange the organs of digestion. Great sympathy is known to exist between the organs of generation and the stomach; if the latter be deranged the former feels a corresponding influence, and the sympathetic nerves are the media by which the change takes place.

Emanations from putrid animal remains, miasmata, overfeeding, derangement of the stomach, stimulating powders containing demulcents and diuretics, blows, excitement, injuries, musty fodder, indigestible food, and a low condition or plethora may cause it.

I was called, several months ago, to a dairy where from twenty to thirty cows (Holstein) are kept—never less than eight or ten cows.

On the last visit I found a young bull in the midst of the herd of cows; also an older bull among them; the younger animal teasing and exciting one cow, then another. I witnessed this, and notified the owner to remove them—to tie up the young animal. This remedied the evil. I had stagnant pools which were in the barnyard, and had caused abortion for years, removed or filled up. After the removal of these pools the cattle improved.

I will relate a case of abortion in a fine setter bitch. The owner explained the case to me, which occurred about the holidays. He could not account for it; the loss of the pups grieved him, as they were valuable. No bad odors, no slaughter-house near—nothing. Questioned him: "I suppose you killed a turkey?" He said, "Yes; just the day before the loss of the pups." I also asked him: "Was the animal present at the time?"

[1] Presented at the annual meeting of the Pennsylvania State Veterinary Medical Association, March 4 and 5, 1902.

"Yes; licked up some of the blood; took the head of the turkey in her mouth; appeared very much excited, running up and down the yard. The next day appeared to have some irritation of the vagina, and in a few hours gave birth to her pups." I think this case worth mentioning.

*Preventives.* Isolation, disinfectants, and fumigants. Give daily some preparation to each cow with calf, for four or five days.

In the majority of cases the placenta must be removed.

